# Clio in the Operating Theatre: Historical Research, Emotional Health, and Surgical Training in Contemporary Britain

**DOI:** 10.1093/jhmas/jrac045

**Published:** 2023-03-04

**Authors:** Agnes Arnold-Forster

**Affiliations:** London School of Hygiene and Tropical Medicine, UK

**Keywords:** Emotions, Surgery, Britain, Training, Professional Engagement, Medical Students

## Abstract

Drawing on my experience working as a postdoctoral research and engagement fellow on the Wellcome Trust-funded project, Surgery & Emotion, this article reflects on this innovative model of historical research and professional engagement, explores the challenges posed by crossing disciplinary boundaries, and interrogates the practical and theoretical utility of bringing historical research into the operating theatre. How do surgeons specifically engage with the history of their profession? What can the history of emotions offer to the training of medical students and surgeons? What obstacles interfere in this type of cross-disciplinary engagement? What peculiar opportunities and challenges do the United Kingdom higher education system and National Health Service pose to the teaching of medical history in clinical settings?

Bringing Clio into the operating theatre provides surgeons with an alternative narrative to that which they have come to expect about the emotions they ought to feel and express in their work. It allows them to explore the high feelings of their professional lives at a remove and offers an array of possible solutions to the current emotional health crisis in British medicine. History allows surgeons to imagine an alternative world: one where the pervasive and persistent models of emotional detachment – damaging to both patient experience and professional wellbeing – dissolve.

The surgical profession has long been associated with emotional detachment, dispassion, and even brutality. Prior to the advent of anesthesia in the 1840s, surgeons operated only infrequently. When they did, they employed little or no pain relief and occasioned great physical agony and emotional distress on themselves and their patients. They acquired reputations as barbarous butchers who cared little for the suffering of their patients.^1^ Despite recent historical research by Michael Brown in particular that demonstrates that nineteenth-century surgeons were in fact profoundly troubled by their patients’ distress, this stereotype still permeates accounts of pre-modern surgery in both academic and lay histories and persisted into the twentieth century.^2^ By the 1950s, the shape of this surgical stereotype had solidified. Authoritarian and autocratic, he (and it was almost always he) was prone to unpredictable outbursts of anger. He cut first, asked questions later, and was never in doubt. He was good at “hard” surgeries, but bad at “soft” skills such as compassion and communication. He operated with dispassion but occasionally his capacity for emotional detachment tipped into cruelty – causing psychological harm to both himself and his patients.

History can offer surgeons an alternative vision of this mythos of professional culture – one that is more emotionally healthy and inclusive – and if this vision is effectively communicated, can challenge pervasive stereotypes and aid surgical recruitment. Between 2017 and 2020, I was postdoctoral research and engagement fellow on Surgery & Emotion.^3^ Surgery & Emotion was an interdisciplinary, four-year project that explored the emotional landscape of surgery from c.1800 to the present day. It was supported by a Wellcome Trust Investigator Award, conceptualized and led by Michael Brown, and was based at the University of Roehampton. This collaborative project examined the place of emotion within the practice, politics and representation of surgery from the nineteenth to the twenty-first century. It brought together historians and literary scholars working on nineteenth-century surgery in both peace and war, as well museum studies researchers investigating the representation of surgical practice and bodily remains in exhibitions.^4^ Our aim was to historicize the conditions of surgical work in the present and to demonstrate the rich emotional lives of surgeons in the past.

My strand of the project blended histories of work, medicine, and the emotions to map out the personal and professional landscape of surgery from the foundation of the National Health Service in 1948 to the present day. Like my colleagues, I sought to problematize stereotypes of surgical dispassion and their place in historical narratives and contemporary culture. My work revealed that contrary to assumptions, emotions were central to the practice and perceptions of twentieth-century surgery. Despite this, feelings play only a limited role in the formal and informal training of medical students and surgeons in modern and contemporary Britain.^5^ I had a dual role on the project. I was both a historian – conducting archival research and oral history interviews – and also an engaged researcher seeking to communicate project findings and effect measurable change.

The goal was to challenge surgical stereotypes by doing three key things. First, to use historical research to demonstrate the diverse array of emotional experiences had by surgeons in the past and offer present-day practitioners (particularly those in training) a broader range of possible temperaments, behaviors, or affective attitudes to adopt. Second, to use history to demonstrate the constructed and contingent nature of surgical dispassion. In other words, to show that the now-prevalent surgical stereotypes are not universal in time and place, but rather a product of specific social, cultural, and political circumstances. Emotional detachment is not, therefore, an essential or innate characteristic that the surgeon must possess or acquire. Its centrality to the surgical identity was made and can, therefore, be unmade. Finally, our professional engagement events also offered surgeons an opportunity to discuss a range of feelings, openly and without fear of professional retribution. One of the advantages of a historical project is that it allows people who might not be accustomed to talking about their own emotions the chance to reflect on the feelings evoked by surgical work at a remove. It allows them to displace discussions about highly intimate, sensitive, or distressing subjects onto historical actors and away from themselves.

In this article, I will begin by outlining the pernicious and problematic consequences of the surgical stereotype on recruitment, retention, and workplace wellbeing. This stereotype has taken shape in twentieth-century cultural representations of surgeons and emerges in oral history interviews I have conducted with practitioners. There is ample evidence drawn from the social sciences and medical education research that demonstrates the lasting impact of this stereotype on medical students’ and trainees’ emotional health, their perceptions of surgeons, and their likelihood of entering or remaining in the profession. From its outset, the project set out to use historical research and engagement activities to address these problems. In what follows, I will outline three of the activities we ran between 2017 and 2020 and demonstrate the impact they had on participants’ perspectives. Finally, I will discuss some of the challenges involved in engaging with surgeons and a non-clinical public with medical history. I argue that this is an emotionally demanding form of academic labour, with potential hazards for the historians involved. It is worth noting that I did almost none of this engagement work alone and that this job involved sustained intellectual and practical collaboration. That being said, the reflections in this article are my own and other members of the project team may not agree with my problems and perspectives.

## EMOTIONS AND THE SURGICAL STEREOTYPE

The surgical stereotype was best embodied by author and anesthetist Richard Gordon’s irascible Sir Lancelot Spratt. Spratt first appeared in Gordon’s 1952 novel, *Doctor in the House* and its many sequels.^6^ A dictatorial demagogue, he strode down hospital corridors with a team of frightened trainees hurrying along behind him. In one iconic scene, Spratt stands at a patient’s bedside firing questions at a gaggle of medical students, one of whom has just examined the hapless and prone sufferer and found a lump. “Is it kidney? Is it spleen? Is it liver? Is it dangerous?” barks Spratt, before drawing a long incision line on the patient’s abdomen, then turning to the by-now highly alarmed patient to say, “Now don’t worry, this is nothing whatever to do with you.”^7^

Sir Lancelot Spratt was and is an archetype. He represented and constructed a lasting and influential caricature that can be traced through fiction, film, and professional discourse from the 1950s to the present. Revealing or demonstrating the “relative throw – the weight or significance” of culture on ordinary people in the past is an unresolved challenge to the cultural historian.^8^ From my oral history interviews and engagement with surgeons, it is clear that while they might be fictional and sometimes even absurd, these stereotypes have shaped the surgical experience in Britain and continue to inform the nature and conditions of surgical identity and work today. One of the most obvious ways that these historical representations continue to influence notions of surgical professionalism is the pervasive notion that surgical care is – to a lesser or greater extent – incompatible with emotions. It was present in mid-century cultural representations of the profession such as *Doctor in the House* and appeared in contemporaneous surgical textbooks. In *The Surgeon’s Craft*, published in 1965, the author answers the self-posed question, “What then is the typical surgeon?” by insisting that he must possess, “a rather more than normal degree of common sense which will enable him in the early stages of his career to inhibit his emotional responses to the sometimes tragic situations which confront him and to get on with the job that needs to be done.”^9^ More than fifty years later, most of the surgeons I have interviewed insisted that they should be emotionally detached from their patients. For example, one plastic surgeon said, “When you’re making decisions and operating on people you’ve got to be detached.”^10^

Evidently, surgeons see emotional detachment as a desirable professional characteristic. Practitioners I interviewed tended to give one of two reasons for the importance of eschewing feelings from surgical practice. One, they argued that emotions have the capacity to contaminate the surgeons’ ability to make decisions, operate effectively, and provide good clinical care. Along these lines, they suggested that if they were to feel too much for their patients, then they would become overly invested, rendered incapable of judging each case equally, and rely on emotions rather than evidence when diagnosing and determining appropriate interventions. Two, they suggested that allowing yourself to become emotionally invested can harm your psychological or emotional health. They claimed that surgeons – like all of us – have finite emotional resources and that those resources need to be protected, maintained, and replenished. As medical anthropologist Jodi Halpern suggests, doctors often insist that detachment is necessary for practitioners to provide “objective” medical care and to avoid “burning out.”^11^ This latter conceptualisation has become increasingly popular, and partly relies on newer ideas about wellness and recent developments in psychological understandings of the emotional burdens of labour.^12^

Both rationales have problematic, if unintended, consequences. Stereotypes of surgical dispassion carry over to the non-clinical public and have been found to dissuade medical students and trainee doctors from entering the profession.^13^ This limits the profession’s diversity, maintains cultural homogeneity, and restricts the range of people who feel that surgery is the right career for them.^14^ In both Britain and North America, applications to surgical specialities since the millennium have seen a significant drop in numbers. In surveys designed to identify why this might be, respondents held uniform stereotypes of surgeons as self-confident and intimidating; surgery was competitive, masculine, and required personal sacrifice.^15^ To succeed, students felt they must fit these stereotypes, excluding those unwilling or unable to conform. For many, therefore, surgery was neither an attractive nor realistic career option. Lasting surgical stereotypes continue to deter students from the specialty.

In addition, assumptions that surgeons’ feelings are best left undiscussed or unexamined could cause emotional harm to practitioners. In a community where emotional detachment is a normative value, a professional culture that valorises resilience and machismo predominates.^16^ This, in turn, means that there are few formal or informal outlets for emotional distress or opportunities for therapeutic support.^17^ Surgery is a profession that makes little space for emotional reflection. This is the case even though surgeons shoulder intense emotional experiences and are expected to deal with complex and sometimes frustrating working lives.^18^ While these sources of frustration in the surgical workplace have attracted new attention from surgeons, professional organizations, and health policymakers, recent studies have revealed a high level of burnout among doctors and medical students in the UK and new and persistent pressures have led to a supposed epidemic in serious psychological and emotional conditions.^19^

Surgery is beset, therefore, with a dual and inextricably linked set of problems. It is an emotionally demanding profession, and practitioners undergo a range of workplace stressors resulting from their conditions of labour. In surgery’s professional tradition, the normative response to emotional difficulties is repression. This is true both culturally and institutionally. It is born out both in the day-to-day interactions between professionals and patients, and in the support and resourcing clinical and educational institutions do or do not provide. These problems require solutions, and solutions are what the Surgery & Emotion project set out to provide.

## THE PROJECT AND ITS ACTIVITIES

As Jennifer Crane has argued, public and professional engagement can function as a “critical methodology” for historians of healthcare and, in our case, deepened our understanding of surgery, health systems, and workplace cultures in contemporary Britain.^20^ And yet, the drive to engage professionals and the public was also a product of the unique conditions of the UK higher education system. Key funding agencies in the medical humanities, as well as the Research Excellence Framework (REF), “incentivize and encourage” both impact and engagement.^21^ The project was responsible for the production of an Impact Case Study for the humanities department’s submission to the REF. Impact Case Studies are discrete programs of public and professional engagement work, based on original research, that can demonstrate tangible impact on the world beyond academia. Their evidence is assessed and graded, and the outcome has financial implications for the supporting university. These implications are partly responsible for a recent flourishing of UK medical humanities projects that center public and professional engagement.^22^

We organized a series of events and activities designed to attract surgeons, allow the public to participate in our historical research, and work together with practitioners and their professional organizations to improve the emotional health of British medical students and surgical trainees. Our events offered those at the outset of their surgical careers opportunities to discuss their emotional experiences away from education and work and to reflect on the constraints of traditional professional cultures and historical stereotypes. In June 2018, we held a workshop at the Royal College of Surgeons of England that brought together students, surgeons, historians, and policymakers to discuss the emotional experiences of surgery, both past and present.^23^ We explored a range of emotional issues affecting surgeons, stress, burnout, bullying, anxiety, doubt, grief, and compassion. We integrated brief, informal presentations on the history of surgery with the experiences and reflections of practitioners, medical educators, and policymakers. We provided plenty of opportunities for discussion to allow delegates to explore the issues raised in the presentations in more depth and to consider the opportunities and challenges in surgical training, practice, and patient care.

The historical presentations offered an alternative vision of surgery’s emotional landscape, particularly in the nineteenth century. Michael Brown argued that while surgeons tend to be thought of as “coolly dispassionate, and those of the past as brusque or even cruel,” his research has shown that in the early 1800s the “image of the emotionally attuned and expressive surgeon” was also prevalent.^24^ He critiqued the “emotional ahistoricity” in Lynda Payne’s *With Words and Knives* that asserts that “physicians, and especially surgeons, have always had to learn some type of detachment.”^25^ He referred to prominent nineteenth-century practitioners such as John Bell and Astley Cooper, whose writings demonstrate the pervasive presence of emotional intersubjectivity in the cultures of early nineteenth-century operative practice.^26^ Not only did Brown’s presentation contradict the claim that surgeons have always had to practice emotional detachment, but also it indicated the contingent nature of the surgical stereotype and temperament. He charted a richly textured account of the changing affective norms of eighteenth- and nineteenth-century surgery – one that complicates sweeping and anachronistic narratives of surgeon’s emotional styles. In doing so, his presentation implied to the audience of twenty-first-century surgeons that they need not conform to stereotype and are free, just like their nineteenth-century predecessors, to remake the emotional landscape of their profession.

Throughout the day, breakout discussions centered on recommendations for change. Much of the conversation focused on how to improve the emotional health of surgeons by making them feel valued and helping them to develop supportive working relationships. Rather than excluding emotions from the professional arena, participants wanted a more “compassionate community” amongst staff. They also recommended alterations and adaptations to the hospital environment. They suggested that forums for socializing, such as doctors’ mess parties and team lunches, would help break down historical hierarchies and boundaries between clinical and managerial staff. They concluded that surgeons need spaces where they can take time to process difficult or emotionally intense events.

Some attendees suggested that emotional health and effective communication should be compulsory elements of continuing professional development rather than “optional add-ons.”^27^ In discussing how to ameliorate grief, anxiety, and doubt, delegates suggested the creation of “Emotional Firms,” “collections of surgeons from different specialties and career stages in different teams who come to work together regularly to talk about the social and emotional aspects of their work.”^28^ Existing Morbidity and Mortality meetings could also be restructured to allow time and space for affective reflections, not just clinical commentary. Finally, recognizing the historically specific nature of emotional experience and expression, they considered how compassion and sympathy might be curtailed by scheduling restraints. Some talked about the challenges of giving patients adequate time to express themselves within brief consultations and reflected that offering care and compassion is a form of emotional labour, one that is too often taken on disproportionately by women.^29^ The importance of collaboration and multi-disciplinary team-working was a major theme coming out of the workshop and this emphasis prompted us to plan a follow-on event that addressed precisely those issues.

In March 2019, we held an event at the Royal College of Nursing to involve more people from the NHS workforce in a dialogue about history and emotional health.^30^ We split the day into two parts. The first session looked at “Experiences from the Operating Theatre,” while the second focused on “Impacts and Solutions.” Each session opened with a panel, featuring informal presentations from surgeons, anesthetists, allied health professionals, and nurses. The presentations were followed by breakout discussions where delegates explored the issues in more depth. Historical research and perspectives were interwoven throughout the day. The sessions were chaired by historians of medicine and healthcare (myself included) who acted as respondents, using historical research to frame the papers dealing with contemporary emotions.

One of the key historical themes of the day was the impact of rigid hierarchies and uneven power structures on operative experience and practitioner emotional health. Jack Saunders, Sarah Chaney, and I looked at the history of hospitals in Britain and identified various moments where individuals and organisations, including trade unions, had attempted to reform the social structure of healthcare provision. These brief responses, based on our research, not only historicized the emotional landscape of the twenty-first-century hospital, but also offered ideas and inspiration for change. My own research into surgical resilience suggests that rhetorical ties between surgery and the military hardens professional hierarchies and fosters a form of emotionally repressed masculinity.^31^ Saunders argued that identifying historical continuities does not mean that problems are unchanging or unfixable. In the breakout sessions, attendees shared and reflected on their own experiences of working in operating theatres. Participants were encouraged to identify challenges they had faced that they wanted to address or overcome. We also gave space for delegates to explore solutions and recommendations for change. These prompts resulted in thought-provoking discussions. Much like at the RCS, key themes included the scarcity of time, the importance of dedicated rest and reflection spaces for staff, and for teams to bond outside the immediate pressures of work.^32^

Running parallel to these workshops for professionals, Michael Brown and the project secured enrichment funding from the Wellcome Trust to develop an ambitious public engagement program. A central strand of this was the “Surgical Speed-Meets,” which we held across the UK (until the COVID-19 pandemic prevented further in-person events).^33^ Our aim in these public-facing activities was to demystify the often-closed world of surgical practice and unpack some stereotypes of surgical detachment – both for the non-clinical audience and for the surgeons themselves. These events enabled surgeons and members of the public to have candid, one-to-one conversations about how it feels to practice and undergo surgery. They followed a “speed-dating” format. We began the evening with short historical presentations from the project team, followed by the Speed-Meets themselves. Michael Brown and I reflected on the implications of our historical research on the nineteenth and twenty-first-centuries respectively on the emotional landscape of surgery today. As in the workshop at the RCS, Brown’s presentation problematized the notion that surgeons have always been, and therefore must be, detached or dispassionate arguing instead for a more emotionally engaged and expressive profession. In my talk, I reflected on the range of different emotions surgeons expressed in my oral history interviews. Rather than focusing on the high feelings of grief, anxiety, and depression I talked about the more mundane emotions that accompany surgical work such as frustration, fatigue, boredom, and contentment. Both presentations set the emotional tenor for the ensuing discussions by cultivating a space in which feelings were at the forefront of participants’ minds. They also used historical examples to delineate a range of different emotional styles and temperaments, complicating popular stereotypes of surgical detachment and provided alternative models for practitioners to follow.

The events consisted of a small group of around thirty participants (fifteen surgeons and fifteen members of the public). Each pair had three minutes to interact before moving on. Each round had a different theme. Upon arrival, participants wrote down one emotion they would like to talk about during the evening. We read out these words to help structure each three-minute session and prompt discussion. Emotions ranged from “curiosity” to “stress,” “elation” to “apprehension.” After the Speed-Meets were complete, our surgeons and public participants re-convened for a group discussion about what they had gained by taking part and whether their perspectives had altered as a result. The conversation also broached a wide range of subjects, including surgeons’ experiences of racism and sexism, patient expectations, team-working, and representations of surgeons in the media and popular culture.^34^

## THE CHALLENGES AND OPPORTUNITIES OF PUBLIC AND PROFESSIONAL ENGAGEMENT

These three activities brought the history of surgery and emotions to bear on contemporary operative practice and surgical training. Feedback collected at the events and in the months following demonstrate the practical and theoretical utility of public and professional engagement. One of the project’s key aims was to use history to problematise assumptions of emotional detachment among surgeons. For reasons outlined above, if surgeons expect or demand emotional detachment from themselves or their colleagues, this can prove problematic for their own emotional wellbeing. My research centers the diverse affective experiences of surgeons, examines their feelings, and interrogates the emotional relationships practitioners formed with patients and colleagues. Bringing Clio into the operating theatre thus gave surgeons space to discuss emotions and to reflect on the affective landscape of their working lives. One trainee surgeon at our first workshop commented that, “it felt productive having time to discuss ideas.”

Another surgeon who attended our first workshop said that they no longer “felt alone” in their experiences, and someone else who attended our second workshop reflected that they now knew it was “normal to feel emotions” at work. Following a digital event we ran in May 2020, one practitioner said, “I always thought that it is professional to not show your emotions. How wrong I was.”^35^ Another practitioner who attended our first Speed-Meet said that participating and hearing about the history of surgery made him “think a little bit more about how I approach patients.”^36^ One surgeon who mentioned he was “very emotional with patients” said, “you don’t treat them as any lesser just because they need you, need your services…What I take home today is that I need to do more of this.”^37^ One reflected that, “If you cannot be emotionally engaged with your patient when they are divulging the pain and suffering they go through on a daily basis, then I think in some respects you have no business being in medicine.”^38^ Asked for their thoughts on the value of historical perspectives on surgery and emotions, one participant at our first workshop testified to the value of history to offer space for emotional reflection, a “contextualised historical account of emotions […] helps to dismantle inaccurate stereotypes and can provide catharsis for those who have struggled with the acceptance and role of positive and negative emotions.”^39^

However, professional engagement of this kind has its limitations. We were a small team with limited resources and capacity. As a result, our events could only attract a relatively small number of participants. Approximately sixty people attended the first workshop, fifty attended the second, and a total of around sixty attended the Speed-Meets in both London and Manchester. Together, this makes up only a small proportion of Britain’s surgical community. Despite these limitations, however, not only were we able to widen our reach through associated publications and social media, but also attendees reported returning to their home institutions and applying what they had learned to staff training. One participant ran a staff support session back at her hospital in the south of England to challenge stereotypes of “machismo and bravado” and worked with nurses and the hospital’s wellbeing lead to provide “increased support and a zero-tolerance stance on bullying and verbal aggression.”^40^

Our events have also helped reshape public perceptions of surgeons and challenged widely held stereotypes that they are detached, dispassionate, even unfeeling. At the end of our first Speed-Meet, we gave feedback forms to all participants to record the event’s impact. Many public participants commented on the surgeons’ candour. Having taken part, one said that she realized surgeons were “just emotional beings…my perception towards surgeons are [now] very very different.”^41^ She added, “I feel quite emotional because some of the answers really touched me.”^42^ Another public participant said that “by seeing surgeons face to face, talking to them, getting a bit more insight about them, and how they…work, how they feel…it’s enhanced the way I trust them.”^43^ Another remarked that, “open discussion worked well, amazing to hear surgeons/healthcare professionals talk so openly.”^44^ One attendee observed, “Surgeons are human beings and have emotions.”^45^

Asked if there was anything they had learned or would do differently after the event, one surgeon reflected on their discovery that “patients want emotion from their surgeons.” One public participant said they would “talk and think about surgeons as humans with feelings, and talk more directly to them.” Another mentioned that they would, “feel able to divulge more to surgeons when being treated.”^46^ Participants were also asked whether the event had changed their opinions on the place of emotions in surgery and healthcare. As Alison Moulds identified, the public articulated the greatest shift in attitudes. One person commented that surgeons, “seem less monolithic now,” while another said that they were, “interested in how emotionally intelligent some of the surgeons were” as it “confounded [their] stereotypes.”^47^ Another attendee reflected, “I definitely have a better opinion of surgeons – I did have the impression they were arrogant – I don’t really know why – but none of them were.” At the beginning and close of the evening, we asked all our participants – both surgeons and members of the public – to respond to a statement: “Surgeons are usually emotionally detached.”^48^ On a sliding scale, they had to rate how far they agreed or disagreed. Reflecting on their experience of participating, one member of the public commented, “the opposite of the stereotype was what came through to me.”^49^

At all our events, participants reflected that one of the problems with a project like ours is that it tends to attract the surgeons who are already interested and invested in improving emotional health and ameliorating working conditions. As I have suggested, the project was well-timed – coinciding with a widespread climate of dissatisfaction in British surgery. This coincidence meant that many surgeons were already engaged in thinking about the place of emotions in training and practice and were primed to participate in our activities and events. The practitioners who perhaps needed to be there the most – the ones who embody the stereotypes we are attempting to challenge – were unlikely to participate. Our attendees were, after all, a self-selecting group. In addition, one of my responsibilities on the project was to recruit surgeons and other healthcare professionals to collaborate with and to attract practitioners to our events. And yet, this had some problematic consequences. Attracting surgeons to the project required me to speak their language and at least claim to buy in to their narratives about their work, even when I did not quite agree. This sat uneasily with my desire to maintain a critical, impartial, historically-informed and academic voice. One of the key principals of the social history of medicine is its capacity to provide robust critique of healthcare and its professionals. Critique and collaboration were occasionally uneasy bedfellows.

However, even for those participants who considered themselves well-versed in the emotional landscape of twenty-first-century surgery, our events offered space for reflection, changed perspectives, and effected tangible impacts. We demonstrated the centrality of emotions in surgical practice and professional identity, irrespective of normative claims about detachment and dispassion. These normative claims were made and thus can be unmade. Specifically, our events confirmed the powerful utility of historical approaches and insights in analyzing and assessing surgical training and cultures. One attendee at our first workshop, when asked about the utility of historical insight said that it showed, “how cultural norms and assumptions have become embedded in the surgical encounter. We have [to] understand these if anything is going to change!”

Despite these successes, public and professional engagement is not easy. The bureaucratic demands of funders and the REF constrained the type of work we could do and limited the questions we could ask of our surgical collaborators. The process of producing and evidencing change required us to ask surgeons about the impact of our work on their attitudes and behaviours without acknowledging the fact that impacts can be felt long after an intervention or event has taken place or that ephemeral effects on culture, experiences, and opinions can be difficult to trace. In addition, these activities are costly, time-consuming, and emotionally draining for all involved.

As a female historian at the outset of my academic career – and as someone with no clinical background – I sometimes struggled to be taken seriously by the surgeons we worked with. Surgery was, and continues to be, a deeply hierarchical profession. Despite recent and recurring efforts to flatten these hierarchies and render healthcare more equitable and democratic, surgeons still work according to a rigid system of seniority, responsibilities, and roles. Gender, class, and race play key if lamentable parts in this system. My role in the surgical hierarchy was unclear and sometimes troubling for the practitioners I spoke to. While I only rarely encountered explicit sexism, or received derogatory responses to my presence and my work, many of the surgeons I encountered subtly slotted me into the category of medical student or junior trainee, even asking me to perform manual or menial clinical tasks when in a hospital setting. Moreover, I was trained in the humanities and work with historical methodologies. On occasion, this posed communication challenges. Surgeons are often self-defined scientists who value quantitative evidence and empiricism. The qualitative research they are familiar with is more likely to be social science than social or cultural history, and they sometimes objected to my selection methods, my interview questions, and the conclusions I drew from my research. They were occasionally sceptical of the project and its engagement attempts and wanted a different, more tangible, set of goals and outcomes.

To address these challenges, and respond to some of the scepticism I encountered, I did two main things. Sometimes I had to learn to translate my humanities research into terminology more appropriate to the social sciences. I had to acquire a new vocabulary, one that made the aims and implications of my research comprehensible to people from different disciplinary backgrounds. In other cases, I made use of some surgeons’ interest in medical history and heritage. I could, sometimes, persuade them to participate in my oral history interviews by appealing to their desire for their careers to be recorded for posterity. I also made connections with practitioners who had trained at hospitals, universities, or medical schools I have attended, worked at, or researched.

In contrast, my position as someone situated outside the healthcare hierarchy also proved productive and even powerful. Uninhibited by the trappings of surgical convention and from associations with specific healthcare institutions and professional societies, I was relatively free to critique the cultures and practices of modern British healthcare in ways unavailable to surgeons with duties to their colleagues, employers, and professional communities. My role as an historian also likely engendered greater candour, openness, and honesty in discussions. As someone unattached to a professional body or healthcare institution, I offered an opportunity to discuss some of the darkest features of surgical life and reflect on their most distressing or ignoble emotions or experiences. Some of the surgeons I interviewed described their feeling of relief at having someone to speak to and suggested that they had never been asked to expose their inner lives in such a way.

Running events likes these – especially since they were so focused on emotions – also requires a blurring of the personal and professional. To engender the kind of ease and familiarity that was required for surgeons to open up to us during interviews and engagement activities, occasionally I revealed details about my personal life and clinical history. This was important, not least to avoid what Jennifer Crane calls the “imposition of hierarchies between ‘researcher’ and ‘researched.’”^50^ This vulnerability does, unfortunately, have consequences – and those consequences are gendered and tied to hierarchies internal to academia. As the recent book edited by Tracey Loughran and Dawn Mannay reveals, historical work requires emotional labour and the history of medicine – which often touches on troubling or intimate personal experiences – is even more emotionally demanding than some other historical sub-fields.^51^ Moreover, as Crane, Mary Morris, and Andrea Davies have all observed, the emotional labour of public engagement “fall along gendered lines” and, as Heather Savigny has argued, the “impact agenda” occasionally exposes women to “structural and symbolic violence.”^52^ These challenges are also, as Crane has observed, made more acute by an academic system “reliant on fixed-term labour” – especially when that fixed-term labour tends to be disproportionately done by people of colour, women, and scholars from working class backgrounds.^53^ While our project was a truly collaborative one, the risks of engagement – emotional and professional – are often born by the precarious or junior scholars planning and running the activities, whereas the benefits are distributed largely to institutions.

## CONCLUSION

There is a palpable sense of crisis among surgeons – one that is being insufficiently met by interventions from government, professional societies, and individual institutions. History offers a range of insights into this current climate of surgical distress. It provides practitioners with an opportunity to rethink not just the past, but also the present and future of surgery. It broadens the profession’s imaginative horizons and enables surgeons to think beyond individualistic responses to workplace dissatisfaction and see current problems in their structural, political, and historical context. It offers surgeons an opportunity to engage with questions of funding, management, and the maintenance of the welfare state. It also historicizes aspects of the surgical identity that might prove problematic – prompting them to see emotional detachment, hierarchy, and authoritarianism as social constructs that need not apply to the twenty-first-century. It provides examples of successful interventions from the recent past such as dedicated hospital rest and social spaces, psychological support, widespread counselling services, and in-built opportunities for reflection that could be adapted to the present day. History allows surgeons to imagine an alternative world, one where the pervasive and persistent models of emotional detachment – damaging to both patient experience and professional wellbeing – dissolve.

Surgeons were not the only ones to benefit from this project. Historians of medicine have much to gain here as well. Public and professional engagement – at least in Britain – is an emerging field of work, one that is increasingly theorized and institutionalized. More and more academic historians will need to acquire the skills necessary to perform such labor effectively. I have left the project with a newfound confidence in my ability to communicate with clinical practitioners, a deeper understanding of the structures of British healthcare and medical education, and a range of practical, event management, social media engagement, and writing skills. I am also convinced that despite its many challenges, engaging healthcare professionals and the public with medical history research has the capacity to transform the professionals’ behaviors and attitudes towards medicine and health in the past, present, and future. But, we must be cautious. The time and labor of those academics we call on to practice this engagement must be protected, supported, and adequately remunerated. It must not be just time away from their work of research and it must be valued by hiring committees. If not, the profession risks losing not only valuable junior scholars – who currently undertake much of this work – but also their expertise.

## Footnotes

1 See Lindsey Fitzharris, *The Butchering Art: Joseph Lister’s Quest to Transform the Grisly World of Victorian Medicine* (London: Penguin, 2017); Agnes Arnold-Forster, “Review of *The Butchering Art: Joseph Lister’s Quest to Transform the Grisly World of Victorian Medicine*,” *Reviews in History* (2018), DOI: 10.14296/RiH/2014/2256.

2 Agnes Arnold-Forster, “Gender and Pain in Nineteenth-Century Cancer Care,” *Gender and History* Online First (2020), https://doi.org/10.1111/1468-0424.12468; Michael Brown, “Surgery and Emotion: The Era Before Anaesthesia,” *The Palgrave Handbook of the History of Surgery*, ed. Thomas Schlich (London: Palgrave Macmillan, 2017), 327-348.

3
www.surgeryandemotion.com

4 For the first two years, I worked closely with my colleague Alison Moulds, who was the project’s engagement fellow before she left to take up another position. Other project colleagues include James Kennaway, Lauren Ryall-Waite, and latterly David Saunders.

5 Agnes Arnold-Forster, “A Small Cemetery: Death and Dying in the Contemporary British Operating Theatre,” *Medical Humanities* 46 (2019): 278-287.

6 Richard Gordon, *Doctor in the House* (London: Michael Joseph, 1952).

7 John Spencer, “Some Activity but Still Not Much Action on Patient and Public Engagement,” *Medical Education* 50 (2016): 3-23, 3.

8 Lesley Scanlon, “White Coats, Handmaidens and Warrior Chiefs: The Role of Filmic Representations in Becoming a Professional,” in *“Becoming” a Professional: An Interdisciplinary Analysis of Professional Learning,* ed. Lesley Scanlon (London: Springer, 2011), 109.

9 Hedley Atkins, *The Surgeon’s Craft* (Manchester: Manchester University Press, 1965), 30-31.

10 Interview with male surgeon born in 1946; interviewed by author, 23 March 2018.

11 Jodi Halpern, *From Detached Concern to Empathy: Humanizing Medical Practice* (Oxford: Oxford University Press, 2010), xxi.

12 Liselotte N. Dyrbye, et al., “Development of a Research Agenda to Identify Evidence-Based Strategies to Improve Physician Wellness and Reduce,” *Annals of Internal Medicine* 166 (2017): 743–744.

13 E. J. R. Hill et al., “Can I Cut It? Medical Students’ Perceptions of Surgeons and Surgical Careers,” *American Journal of Surgery* 208 (2014): 860-867.

14 Ibid.

15 Ibid.

16 Alicia J. Polachek, et al., “The Look and Feel of Resilience: A Qualitative Study of Physicians’ Perspectives,” *Journal of Hospital Administration* 5 (2016): 47; Agnes Arnold-Forster, “Resilience in Surgery,” *British Journal of Surgery* (2020); “Doctors’ Wellbeing: Learning from the Past Can Help Improve the Future,” *British Medical Journal* (2018).

17 Arnold-Forster, “A Small Cemetery,” 284.

18 Ibid.

19 British Medical Association, “Supporting Health and Wellbeing at Work,” (2018), https://www.bma.org.uk/media/2076/bma-supporting-health-and-wellbeing-at-work-oct-2018.pdf.

20 Jennifer Crane, “The NHS…Should not be Condemned to the History Books’: Public Engagement as a Method in Social Histories of Medicine,” *Social History of Medicine,* Online First https://doi.org/10.1093/shm/hkaa041.

21 And, medical practitioners are “influenced by the…practices of “public-patient involvement,” which are encouraged by health funders,” Ibid.

22 Ibid.

23 Agnes Arnold-Forster and Alison Moulds, “Surgery and Emotional Health,” *Bulletin of the Royal College of Surgeons* (2018), https://doi.org/10.1308/rcsbull.2018.265; Agnes Arnold-Forster and Alison Moulds, “Operating with Feeling: A Workshop on Surgery and Emotion,” *Surgery & Emotion Blog* (July 2018), http://www.surgeryandemotion.com/blog/view/operating-with-feeling-workshop.

24 Michael Brown, “Surgery and Emotion: The Era before Anaesthesia,” in *Palgrave Handbook of the History of Surgery*, 330.

25 Ibid.

26 Ibid., 334.

27 Arnold-Forster and Moulds, “Operating with Feeling.”

28 Ibid.

29 Ibid.

30 Agnes Arnold-Forster and Alison Moulds, “Emotions and Team-Working in the Hospital: Workshop with the RCN,” *Surgery & Emotion Blog* (March 2019), http://www.surgeryandemotion.com/blog/view/emotions-and-team-working-in-the-hospital-workshop-with-the-rcn.

31 Arnold-Forster, “Resilience in Surgery.”

32 Arnold-Forster and Moulds, “Emotions and Team-Working in the Hospital.”

33 Alison Moulds, “Surgical Speed-Meets,” *Bulletin of the Royal College of Surgeons* (2019): 268-270, https://publishing.rcseng.ac.uk/doi/full/10.1308/rcsbull.2019.268.

34 Alison Moulds, “Our First Surgical Speed-Meet Engagement Event,” *Surgery & Emotion Blog* (August 2019), http://www.surgeryandemotion.com/blog/view/our-first-surgical-speed-meet-engagement-event.

35 Event feedback.

36 Moulds, “Surgical Speed-Meets,” 270.

37 Ibid.

38 Event feedback.

39 Arnold-Forster and Moulds, “Operating with Feeling.”

40 Agnes Arnold-Forster and Alison Moulds, “Emotions and Team-Working in the Hospital: Workshop with the RCN,” *Surgery & Emotion Blog* (March 2019), http://www.surgeryandemotion.com/blog/view/emotions-and-team-working-in-the-hospital-workshop-with-the-rcn.

41 Alison Moulds, “Our First Surgical Speed-Meet Engagement Event.”

42 Ibid.

43 Ibid.

44 Ibid.

45 Ibid.

46 Ibid.

47 Ibid.

48 Ibid.

49 Ibid.

50 Crane, “The NHS…Should not be Condemned to the History Books,” 18.

51 Tracey Loughran and Dawn Mannay (eds.), *Emotion and the Researcher: Sites, Subjectivities, and Relationships* (Bingley: Emerald Publishing Limited, 2018).

52 Mary Morris and Andrea Davies, “Being Both Researcher and Subject: Attending to Emotion within Collaborative Inquiry,” in *Emotion and the Researcher* 229–244; Heather Savigny, “The Violence of Impact: Unpacking Relations Between Gender, Media and Politics,” *Political Studies Review,*https://doi.org/10.1177/1478929918819212. See also Crane, “The NHS…Should not be Condemned to the History Books.”

53 Crane, “The NHS…Should not be Condemned to the History Books,” 22.

